# Active Smoking Before Liver Transplantation in Patients with Alcohol Use Disorder: Risk Factors and Outcomes

**DOI:** 10.3390/jcm9092710

**Published:** 2020-08-21

**Authors:** Ana Isabel López-Lazcano, Antoni Gual, Jordi Colmenero, Elsa Caballería, Anna Lligoña, Miquel Navasa, Gonzalo Crespo, Eva López, Hugo López-Pelayo

**Affiliations:** 1 Grup Recerca Addicions Clínic (GRAC-GRE), Department of Psychiatry, Clinical Institute of Neuroscience, Hospital Clínic i Universitari de Barcelona, Universitat de Barcelona, IDIBAPS. RTA (RETICS). Villarroel, 170, 08036 Barcelona, Spain; tgual@clinic.cat (A.G.); caballeria@clinic.cat (E.C.); alligona@clinic.cat (A.L.); HLOPEZ@clinic.cat (H.L.-P.); 2 Liver Unit, Hospital Clínic i Universitari de Barcelona, Universitat de Barcelona, IDIBAPS. CIBERehd. Villaroel 170, 08036 Barcelona, Spain; jcolme@clinic.cat (J.C.); mnavasa@clinic.cat (M.N.); gcrespo@clinic.cat (G.C.); elopezb@clinic.cat (E.L.)

**Keywords:** smoking, alcohol use disorder, liver transplant, risk factors, survival

## Abstract

Tobacco use is more prevalent among alcohol liver disease (ALD) transplant patients and exerts harmful effects to the patient and to the graft. The aims of this study were to examine the impact of smoking status (nonsmoker, ex-smoker, active smoker) on patient survival and clinical outcomes, and to assess risk factors for active smoking before and after liver transplant (LT). An observational retrospective cohort study with 314 ALD patients undergoing LT from January 2004 to April 2016. Recipients were followed until April 2017 or death. Kaplan–Meier and Cox proportional hazards regression analyses were used to assess risk of mortality according to smoking status before LT. Smokers had a 79% higher risk of dying than those who had never smoked or quit smoking before LT. Ex-smokers had a greater survival probability (96.2%, 93.8%, 86.9%, and 83.1% at 1, 3, 5, and 10 years after LT) than active smokers until LT (96.0%, 85.6%, 80.0%, and 70.4%). Active smokers before LT with poor toxicity awareness had more than a twofold higher risk of mortality (Cox HR = 2.20, 95% CI: 1.05–4.58, *p* = 0.04) than ex-smokers. Younger age (OR = 94), higher Model for End-Stage Liver Disease (MELD) (OR = 1.06), and comorbid substance use disorder (OR = 2.35) were predictors of smoking until LT. Six months or less of alcohol abstinence (OR = 3.23), and comorbid substance use disorder (OR = 4.87) were predictors of active smoking after LT. Quitting smoking before transplantation improved survival. Evidence based smoking cessation interventions should be offered before and after LT.

## 1. Introduction

Smoking is a major public health concern and a preventable cause of morbid-mortality, with more than eight million deaths a year worldwide attributable to tobacco [1]. Tobacco use increases the risk of post-surgical complications, such as impaired heart and lung functions. It affects the immune system, delaying or impairing wound healing and increasing the risk of infections. Quitting tobacco four weeks before surgery is associated with less postoperative complications and each additional week of abstinence improves health outcomes [2].

There is increasing evidence suggesting that smokers have higher risk of liver cirrhosis regardless of alcohol use, and that alcohol and tobacco have a more hazardous and synergistic effect when they occur together [3,4]. Tobacco use is more prevalent among patients with alcohol liver disease, with an estimated prevalence of 52% and 44% of active smokers before and after liver transplantation (LT), respectively [5]. Alcohol liver disease (ALD), normally associated with alcohol use disorder (AUD), is estimated to contribute up to 50% of the overall cirrhosis burden in the United States and worldwide [6]. According to the World Health Organization, 73.8% of liver cirrhosis among men and 56.3% among women can be attributed to alcohol in Spain [7].

Tobacco is a risk factor for cardiovascular events and cancer, which are among the main causes of death of LT patients [8,9]. There is evidence of the harmful effects derived from smoking to the patient (de novo malignancy, cardiovascular disease) and to the graft (hepatic artery thrombosis, biliary complications) [10]. Even though some patients stop smoking before LT, many of them relapse early after it, increasing their use over time [11,12], with alcohol relapse higher in smokers [10].

In the setting where this study was conducted, tobacco use was not an absolute contraindication for liver transplantation, although abstinence was strongly encouraged [13]. All the patients with alcohol-related cirrhosis were evaluated before and after LT for alcohol consumption, tobacco use, and substance abuse. Therefore, this cohort represents a unique opportunity to assess the effects of tobacco in liver transplant recipients.

The aim of the present study was to assess the impact of smoking status before LT (nonsmoker, ex-smoker, and active smoker) on patient survival, as well as on clinical events (alcohol and substance use relapse, graft cirrhosis, biopsy-proven alcoholic steatohepatitis, recurrence of hepatocarcinoma, skin cancer, and nonskin cancer) during follow-up. A secondary aim of the study was to assess risk factors associated with active smoking before and after LT. We hypothesized that active smoking before LT could be associated with higher mortality and worse outcomes after LT.

## 2. Materials and Methods

### 2.1. Participants and Procedure

This observational retrospective cohort study was carried out with data from a previous study that assessed risk factors for alcohol relapse in a cohort of patients (recruitment flowchart in supporting material, Figure S1) undergoing LT from January 2004 to April 2016 at Hospital Clinic of Barcelona, Spain [14]. All recipients were followed until April 2017 or death. Primary sources of information were reviewed for this article.

Data collected, as well as psychiatric and medical evaluation and post-transplant follow-up procedures (supporting material), were the same as in the previous study related to alcohol relapse [14]. At baseline, collected data included demographic characteristics, etiology and severity of liver disease, hepatocellular carcinoma (HCC), donor type, serological status of human immunodeficiency virus (HIV), alcohol and substance screening (cannabis, cocaine, opioids, benzodiazepines, and nicotine), and psychosocial history.

The follow-up assessment included alcohol relapse (date, amount and type of drinking), concomitant substance use relapse, tobacco use, cirrhosis of the graft by elastography and/or biopsy, patient survival, and causes of death. Patients were evaluated by a psychiatrist and a clinical psychologist before LT following a well-defined protocol [13]. Abstinence of alcohol and other substances was screened regularly with urine tests, and tobacco abstinence using self-reports. During the time patients were on the waiting list, patients with AUD received individualized treatment. After LT, patients were followed by a hepatologist and were referred to their local Addiction Unit.

Smoking status was categorized into “nonsmokers,” “ex-smokers,” and “active smokers.” During the psychiatric assessment before LT, participants were asked whether they were smoking tobacco regularly at the time of the evaluation, and those who gave an affirmative answer were classified as “active smokers.” Nonactive smokers were also asked whether they had ever smoked tobacco regularly but were not smoking at the present time. Those who provide an affirmative answer and reported abstinence within the last 30 days were categorized as ex-smokers. Those who gave a negative answer to the previous questions comprised the “nonsmokers” group. Although, in some patients, cotinine values were analyzed in urine controls, for the majority of the sample, smoking behavior was self-reported.

Liver biopsies were performed as dictated by clinical circumstances—usually to investigate unexplained derangement of liver biochemistry and/or suspicion of rejection. Transient elastography was routinely performed during follow-up since 2004. All the patients had a liver biopsy or a transient elastography performed within the last year of follow-up. Graft cirrhosis was defined by transient elastography values higher than 14.5 kPa [15] or liver biopsy showing severe fibrosis (F4) or established cirrhosis. No specific cancer detection protocols were applied, and information was gathered only through interview and medical history review.

### 2.2. Data Analysis

The SPSS, version 20.0 (SPSS, Inc., Chicago, IL, USA) statistical package was used to perform the survival analysis and to assess the predictive value of clinical variables on smoking status, with a 95% confidence interval and *p* values.

Kaplan–Meier analysis with log-rank test was used to assess patient and graft survival depending on smoking status (active versus past smokers and nonsmokers) before LT. Cox proportional hazards regression analysis was performed to assess the risk of mortality according to the smoking status before LT. Results were presented as hazard ratios (HR) with 95% confidence intervals (CI). It was considered significant a *p* value of less than 0.05. Follow-up was recorded up to 10 years after baseline, or until April 2017. Survival was determined as the time in days between the liver transplant and the patient’s “death” or “alive” status.

To assess clinical events after LT (alcohol use relapse, substance use recidivism, graft cirrhosis, biopsy-proven alcoholic steatohepatitis, recurrence of hepatocarcinoma, skin cancer, and nonskin cancer) associated with smoking status before LT, a multinomial logistic regression was used.

Means and standard deviations for continuous variables and percentages for categorical variables were used. Chi square, one-way ANOVA and t-test were used to assess which variables were associated with a higher probability of active smoking before and after LT. Pretransplant risk factors independently associated with being an active smoker before and after LT were assessed using a stepwise binomial logistic regression.

### 2.3. Ethical Issues

The study was approved by the Hospital Clinic Ethical Committee (HCB/2016/0806) and was performed according to the Helsinki Declaration (Fortaleza, Brazil, October 2013), and the Spanish national regulations of biomedical research (Ley 14/2007, July 3). The anonymity of participants and confidentiality of data were guaranteed.

## 3. Results

**Figure 1:**
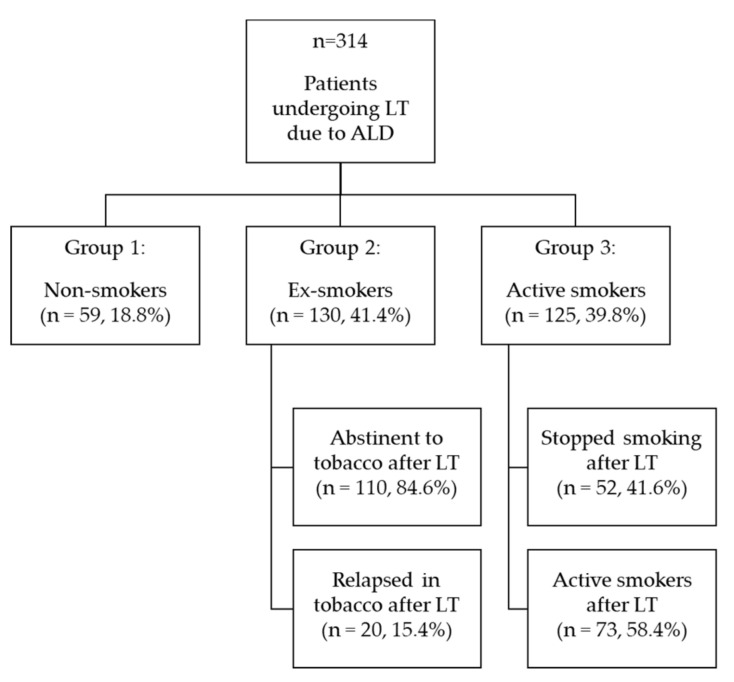
Flowchart of smoking status before and after liver transplant (LT). ALD = Alcohol liver disease.

A total of 314 candidates underwent LT. Smoking status before and after LT is summarized in a flowchart (Figure 1).

At the time of the psychiatric evaluation before LT, 59 patients (18.8%) were nonsmokers (group 1), 130 (41.4%) were ex-smokers (group 2), and 125 (39.8%) were active smokers (group 3). There were 36 (11.5%) patients that abused more than one substance before LT. Baseline characteristics according to smoking status group are described in Table 1.

A total of 283 patients (90.1% of the sample) were male, with no significant differences in gender between groups. Mean age was 55.5 ± 7.5 years (range 25–69), with statistically significant differences between groups as determined by one-way ANOVA (F (2, 311) = 12.86, *p <* 0.0001). An LSD post hoc test showed active smokers were significantly younger (53.0 ± 7.6) than ex-smokers (57.5 ± 6.7, *p <* 0.0001) and nonsmokers (56.3 ± 7.5, *p* = 0.004). Likewise, the mean Model for End-stage Liver Disease (MELD) score was 16.9 ± 6.5, with statistically significant differences between groups (F (2, 311) = 3.14, *p* = 0.044). The MELD score significantly lower for ex-smokers (15.8 ± 6.0) than for nonsmokers (18.0 ± 7.2, *p* = 0.029), and active smokers (17.4 ± 6.5, *p* = 0.049). Younger age, Hepatitis C virus (HCV), unstable family support, and concomitant substance abuse (cannabis and cocaine) were significantly more frequent in patients who were active smokers (group 3) compared to the other groups (Table 1).

### 3.1. Smoking Status at LT and Outcomes

**Figure 2:**
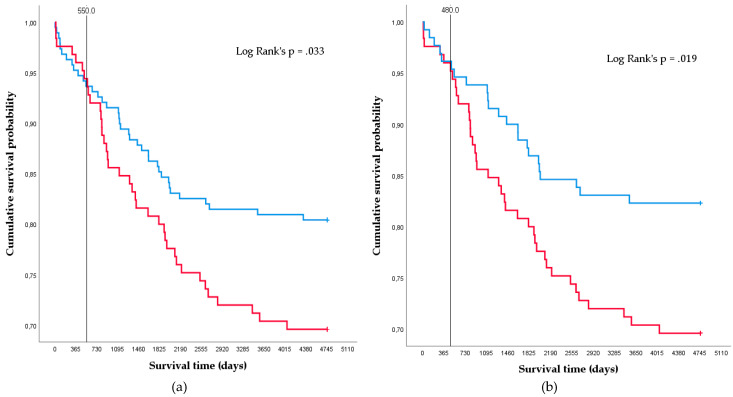
Impact of the smoking status before LT on patient survival. Kaplan–Meier. (**a**) Cumulative survival probability of active smokers (group 3, red line) vs. nonsmokers + ex-smokers (groups 1 + 2, blue line) before LT. (**b**) Cumulative survival probability of active smokers (group 3, red line) vs. ex-smokers (group 2, blue line) before LT.

Kaplan–Meier survival curves are showed in Figure 2. A total of 75 (23.9%) transplanted patients died during a median follow-up period of 10.8 years (5 patients died within the first 30 days, the remaining with a follow-up of 2.5 months–13.0 years). The mortality of the entire cohort was 4.5%, 11.5%, 18.8%, and 23.2% at 1, 3, 5, and 10 years. The number of deaths were 14 (18.7%) among nonsmokers, 23 (30.7%) among ex-smokers, and 38 (50.7%) among active smokers.

Patients who had never smoked or quit smoking before LT showed a greater survival probability than active smokers. The survival probability of nonsmokers and ex-smokers was 95.2%, 91.5%, 85.2% and 81.0% at 1, 3, 5, and 10 years follow-up, which was significantly higher (log-rank *p* = 0.033) than the probability of active smokers 96.0%, 85.6%, 80.0%, and 70.4% (Figure 2a). This difference began to show up 1.5 years after the transplant.

This difference remained significant (log-rank *p* = 0.019) even when comparing only ex-smokers (96.2%, 93.8%, 86.9%, and 83.1% at 1, 3, 5, and 10 years) against active smokers before LT (Figure 2b), showing up 1.3 years after the transplant.

A logistic regression analysis revealed that being an active smoker (OR 1.79, 95% CI: 1.06–3.03, *p* = 0.03) was associated with a higher risk of mortality, with active smokers having a 79% higher risk of dying than those who had never smoked or quit smoking before LT.

When censoring the Kaplan–Meier analyses at three years, these differences remained (log-rank *p* = 0.048 between active smokers and the rest of the sample and log-rank *p* = 0.015 between active smokers and ex-smokers). No significant differences were found at one or five years, although a tendency was observed.

There were not significant differences between groups in graft survival time.

Regarding the results of Cox proportional hazards regression analysis, active smokers had a significantly higher risk of mortality (Cox model 1: HR = 1.63, 95% CI: 1.04–2.56, *p* = 0.034) compared to the rest of the sample. Adjustment for HCV showed similar results (Cox model 2: HR = 1.70, 95% CI: 1.07–2.72, *p* = 0.03).

Likewise, active smokers had a significantly higher risk of mortality (Cox model 3: HR = 1.84, 95% CI: 1.10–3.09, *p* = 0.02) compared to ex-smokers. Adjustment for alcohol toxicity awareness showed that patients who were active smokers before LT and that had a poor toxicity awareness had more than a twofold higher risk of mortality (Cox model 4: HR = 2.20, 95% CI: 1.05–4.58, *p* = 0.04).

As for morbidity during the follow-up period, active smoking before LT was associated with higher risk of recidivism in substance use (*p* = 0.02) and with higher risk of non-skin cancer (*p* = 0.04) (see Table 2).

After LT, nine patients relapsed on substance use (six patients relapsed on cannabis, two patients on cocaine, and one on cocaine, amphetamines, and LSD). Data regarding patients that relapsed on alcohol use after the intervention were published elsewhere [14].

### 3.2. Factors Associated With Active Smoking Before LT

Sociodemographic and clinical variables according to smoking status were compared between active smokers and patients that had quitted smoking before LT to study the factors associated with increased risk of smoking until LT.

Younger age (*p* = 0.00), higher MELD score (*p* = 0.04), presence of hepatitis C virus (*p* = 0.00), having attended more than one alcohol treatment (*p* = 0.03), and having a comorbid substance use disorder (*p* = 0.00) were associated with a higher risk of active smoking until LT. Specifically, those candidates with cannabis (*p* = 0.03) or cocaine (*p* = 0.00) use disorder were more likely to smoke until LT. Liver-renal transplant (*p* = 0.02), good family support (*p* = 0.03), and good alcohol toxicity awareness (*p* = 0.03) were associated with a reduced risk of smoking until LT instead (Table S1 supplementary material).

Using a binomial logistic regression with the variables that showed significance to assess the risk of being an active smoker until LT, younger age with an OR of 0.94 (95% CI: 0.89–0.99; *p* = 0.01), higher MELD score with an OR of 1.06 (95% CI: 1.01–1.12; *p* = 0.03) and having a comorbid substance use disorder with an OR of 2.35 (95% CI: 1.11–4.96; *p* = 0.03) were independent predictors of active smoking until LT (Table 3).

### 3.3. Factors Associated With Active Smoking After LT

During follow-up, 15.4% of ex-smokers (group 2) relapsed after LT, whereas 41.6% of those who were active smokers stopped smoking after LT. During the follow-up, 59 patients (18.8%) were nonsmokers, 162 (51.6%) ex-smokers, and 93 (29.6%) active smokers.

Variables associated with continuing to smoke after LT or relapsing having quitted tobacco before LT were younger age (*p* = 0.00), 6 months or less of abstinence from alcohol (*p* = 0.03), average alcohol intake of more than 20 standard drink units (1 SDU = 10 gr alcohol) (*p* = 0.04), having a comorbid substance use disorder (*p* = 0.00), specifically having a cannabis use disorder (*p* = 0.01), and having unstable family support (*p* = 0.01). On the contrary, patients with HCV (*p* = 0.01) who had abstained from alcohol for more than a year (*p* = 0.02), having good family support (*p* = 0.02) and having a good alcohol dependence awareness (*p* = 0.04) were more likely to remain abstinent from tobacco after LT (Table S2 supplementary material).

Using a binomial logistic regression to assess who had a higher risk of being an active smoker after LT, we found that six months or less of alcohol abstinence with an OR of 3.2 (95% CI: 1.19–8.78; *p* = 0.021) and having a comorbid substance use disorder with an OR of 4.9 (95% CI: 2.17–10.96; *p* = 0.001) were independent predictors of active smoking after LT. Alcohol dependency awareness was a protective factor against smoking after LT, with an OR of 0.43 (95% CI:0.19–0.97; *p* = 0.042) (Table 3).

## 4. Discussion

The results of this study show that smoking is associated with a higher risk of mid- and long-term mortality, finding that smokers had a 79% higher risk of dying when compared to the rest of the sample. Quitting smoking before LT is also a protective factor against mortality. These results support the pertinence of implementing a specialized intervention for smoking cessation integrated with the treatment of alcohol dependence during the pretransplant period. The differences in mortality could be partially explained by the negative outcomes associated to smoking, such as higher risk of nonskin cancer and of recidivism in substance use. The prevalence of smokers in our sample (39.8% before LT and 29.6% after LT) is comparable to that found in other studies about liver transplantation in ALD [11,16].

Smoking cessation interventions should focus on patients with risk factors associated with smoking before LT (younger age, higher MELD score, comorbid substance use disorder), as well as after LT (six months or less of alcohol abstinence and comorbid substance use disorder). In addition, good alcohol dependence awareness was a protective factor from smoking after LT and alcohol relapse was less prevalent among patients who did not smoke after LT [14], evidencing the close relationship between both substances.

Some studies support the relationship between smoking and higher mortality [17,18], although evidence is inconclusive [19]. The current study emphasizes that smoking is related to worse LT outcomes. Similar to our findings, there is evidence that smoking history correlates with development of cancer after LT [8,9,20,21,22], especially among ALD patients, with the joint use of alcohol and tobacco having higher carcinogenic effects [23,24].

Consistent with our findings, ALD patients with comorbid substance use before LT were more likely to continue smoking [11]. These patients need to make a greater effort to stop using other substances in addition to alcohol and may consider continuing to smoke as a minor evil. Some even express the belief that smoking protects them from other addictions that they consider more harmful, even though the evidence is just the opposite [25].

Smoking cessation may reduce the risk of mortality and adverse outcomes. However, most transplant programs do not consider tobacco use as an absolute contraindication for LT [26] because, for a patient with advanced liver disease, this decision may lead to death in the short term. Decisions about suitability for transplantation should be taken by a multidisciplinary team, considering each individual case. During the post-transplant period, the frequent follow-up visits can be regarded as an opportunity to implement smoking cessation programs for those patients that were not able to attain tobacco abstinence before LT, along with interventions focused on preventing alcohol relapses and enhancing other health behaviors such exercise or a balanced diet.

Some limitations can be noted. To begin, this study did not evaluate if smoking after transplantation impacts survival or other clinical outcomes. Further research is needed to assess differences in morbidity and mortality risk depending on the severity of tobacco relapse (number of cigarettes, years of dependence, and scores of severity using structured measures such as the Fagerström Nicotine Dependence test), since this information was not gathered in the study. For the majority of patients, smoking behavior was self-reported, so results could be biased. Comparing the survival of active smokers and nonsmokers, no significant differences were found. The small sample size of the nonsmokers group (*n* = 58) and the small prevalence of the event “dead” in all groups (14 nonsmokers, 23 ex-smokers and 38 active smokers) made it difficult to observe differences in survival.

Despite these limitations, there are several strengths to mention. The study had a long follow-up and a low dropout rate. This sample consisting of ALD patients allowed us to study a population with higher smoking prevalence compared to other indications for LT. Participants were recruited from clinical practice and not for research purposes, which allowed generalization of results. In our center, patients were closely and frequently monitored by the hepatologist after transplantation, providing greater reliability in data collection concerning relapses in tobacco, alcohol, or substances.

## 5. Conclusions

Quitting tobacco before LT impacts survival. Smokers may benefit from targeted interventions, and these should be especially aimed at those who are at higher risk of continuing to smoke until LT (young patients with higher MELD score and substance use disorders) and after LT (patients with substance use disorder, less than six months of alcohol abstinence before LT and poor alcohol dependency awareness). After LT, relapse prevention must not only include alcohol, but also tobacco and other substances.

## Figures and Tables

**Table 1:** Baseline characteristics according to smoking status groups before liver transplant (LT). One-way ANOVA test for continuous variables (age and MELD) and Chi-square for categorical variables.

	Total (*n* = 314)	Group 1: Non-Smokers (*n* = 59)	Group 2: Ex-Smokers (*n* = 130)	Group 3: Active Smokers (*n* = 125)	*p*
Male	283 (90.1%)	50 (17.7%)	119 (42.0%)	114 (40.3%)	0.30
Age, Years (SD)	55.48 (7.47)	56.31 (7.53)	57.50 (6.67)	53.00 (7.59)	0.00
MELD (SD)	16.89 (6.50)	18.05 (7.15)	15.83 (6.03)	17.43 (6.54)	0.04
Liver-Kidney	11 (3.6%)	2 (18.2%)	8 (72.7%)	1 (9.1%)	0.07
HCV	127 (40.4%)	17 (13.4%)	43 (33.9%)	67 (52.8%)	0.00
HBV	13 (4.1%)	2 (15.4%)	5 (38.5%)	6 (46.2%)	0.88
HCC	130 (41.4%)	21 (16.2%)	58 (44.6%)	51 (39.2%)	0.49
HIV	12 (3.8%)	2 (16.7%)	6 (50.0%)	4 (33.3%)	0.83
Good Family Support	261 (86.1%)	54 (20.7%)	115 (44.1%)	92 (35.2%)	0.02
Unstable Family Support	41 (13.6%)	4 (9.8%)	14 (34.1%)	23 (56.1%)	0.03
Living Donor	22 (7.1%)	4 (18.2%)	9 (40.9%)	9 (40.9%)	0.99
Alcohol Abstinence Before LT 1–C6 Months	60 (19.1%)	12 (20.0%)	21 (35.0%)	27 (45.0%)	0.52
Alcohol Abstinence Before LT 7–12 Months	88 (28.0%)	20 (22.7%)	33 (37.5%)	35 (39.8%)	0.48
Alcohol Abstinence Before LT >12 Months	166 (52.9%)	27 (16.3%)	76 (45.8%)	63 (38.0%)	0.21
DHD > 25 Years	107 (34.3%)	16 (15.0%)	51 (47.7%)	40 (37.4%)	0.18
DHD 11–25 Years	161 (51.6%)	32 (19.9%)	65 (39.8%)	64 (40.4%)	0.90
DHD < 11 Years	44 (14.1%)	11 (25.0%)	12 (27.3%)	21 (47.7%)	0.13
SDU < 11	144 (46.3%)	36 (25.0%)	54 (37.5%)	54 (37.5%)	0.04
SDU 11–20	125 (40.2%)	16 (12.8%)	60 (48.0%)	49 (39.2%)	0.04
SDU > 20	42 (13.5%)	7 (16.7%)	14 (33.3%)	21 (50.0%)	0.35
0 Alcoholism Treatment	268 (85.4%)	49 (18.3%)	111 (41.4%)	108 (40.3%)	0.83
1 Alcoholism Treatment	37 (11.8%)	9 (24.3%)	18 (48.6%)	10 (27.0%)	0.23
2 Alcoholism Treatments	9 (2.9%)	1 (11.1%)	1 (11.1%)	7 (77.8%)	0.05
HRAR Score >3	107 (34.4%)	16 (15.0%)	48 (44.9%)	43 (40.2%)	0.36
Concomitant Substance Abuse	92 (29.3%)	5 (5.4%)	31 (33.7%)	56 (60.9%)	0.00
Benzodiazepines	14 (4.5%)	1 (7.1%)	5 (35.7%)	8 (57.1%)	0.32
Cannabis	49 (15.6%)	3 (6.1%)	17 (34.7%)	29 (59.2%)	0.00
Heroin	28 (8.9%)	3 (10.7%)	11 (39.3%)	14 (50.0%)	0.39
Cocaine	51 (16.2%)	3 (5.9%)	15 (29.4%)	33 (64.7%)	0.00
Other	5 (1.6%)	0 (0.0%)	1 (20.0%)	4 (80.0%)	0.17
Toxicity Awareness	264 (84.1%)	53 (20.1%)	114 (43.2%)	97 (36.7%)	0.04
Dependency Awareness	165 (59.1%)	31 (18.8%)	66 (40.0%)	68 (41.2%)	0.91
Psychiatric Pathology	46 (14.6%)	8 (17.4%)	17 (37.0%)	21 (45.7%)	0.68
HADS Depression (Positive)	21 (10.8%)	2 (9.5%)	7 (33.3%)	12 (57.1%)	0.16
HADS Anxiety (Positive)	36 (18.5%)	4 (11.1%)	19 (52.8%)	13 (36.1%)	0.68
Maladaptive Personality Traits	25 (8.1%)	5 (20.0%)	8 (32.0%)	12 (48.0%)	0.56

HCV = Hepatitis C virus; HBV = Hepatitis B virus; HIV = human immunodeficiency virus; HCC = hepatocellular carcinoma; MELD = Model for End-stage Liver Disease; DHD = duration of heavy drinking; SDU = standard drink unit = 10 gr alcohol; HRAR = High-Risk Alcoholism Relapse Scale; HADS = Hospital Anxiety and Depression Scale.

**Table 2:** Impact of smoking status before LT (active smoker, ex-smoker, or nonsmoker) on clinical events during follow-up. Multinomial regression.

	Group 1: Non-Smokers (*n* = 59)	Group 2: Ex-Smokers (*n* = 130)	Group 3: Active Smokers (*n* = 125)	*p*
Alcohol Use Relapse	10	25	35	0.13
Substance Use Relapse	1	0	8	0.02
Graft Cirrhosis	10	17	18	0.77
Biopsy-Proven Alcoholic Steatohepatitis	2	2	1	0.52
HCC Recurrence	3	3	6	0.33
Skin Cancer	2	4	3	0.92
Non–Skin Cancer	2	14	18	0.04

HCC = hepatocellular carcinoma.

**Table 3:** Risk factors of being an active smoker until and after LT using binomial logistic regression.

	OR (95% CI)	*p*
Higher Risk of Being an Active Smoker Until LT		
Age	0.94 (0.89–0.99)	0.01
MELD	1.06 (1.01–1.12)	0.03
Comorbid Substance Use Disorder	2.35 (1.11–4.96)	0.03
Higher Risk of Being an Active Smoker After LT		
Abstinence Period Of 6 Months or Less	3.23 (1.19–8.78)	0.02
Comorbid Substance Use Disorder	4.87 (2.17–10.96)	0.00
Alcohol Dependency Awareness	0.43 (0.19–0.97)	0.04

## Supplementary Materials

The following are available online at https://www.mdpi.com/2077-0383/9/9/2710/s1, Protocol of psychiatric evaluation, Follow-up survey, Recruitment flow-chart; Figure S1: recruitment flow-chart; Table S1. Factors associated with an increased risk of smoking until LT. Student-T test for continuous variables (age and MELD) and Chi-square for categorical variables; Table S2. Factors associated with an increased risk of smoking after LT. Comparison between patients that were active smokers after LT and those abstinent to tobacco after LT. Student-T test for continuous variables (age and MELD) and Chi-square for categorical variables.
