# Supplementary material for: Active Smoking Before Liver Transplantation in Patients with Alcohol Use Disorder: Risk Factors and Outcomes
Source: J Clin Med. 2020 Aug 21;9(9):2710. doi: 10.3390/jcm9092710 (PMC7564808; doi:10.3390/jcm9092710)
Supplement: Supplementary file 1 [file jcm-09-02710-s001.pdf]

Protocol of psychiatric evaluation: Patients were evaluated as candidates for liver transplantation by the addiction psychiatrist according to the following protocol

- Evaluation of sufficient social and family support based on family teamwork, level of knowledge and understanding, toxicity dependency and awareness, consistency in the information given by the patient, family and clinical records.
- Six months of abstinence from alcohol prior to listing, except in patients in whom the hepatic reserve or transplant indication did not allow for this interval of abstinence (patients with MELD > 19).
- Abstinence from other substances including: 1) Opioids, cocaine, hallucinogens and inhalants: abstinence should be of one year or more. If a sporadic episode of relapse had occurred, an additional 6-month period of abstinence after the episode was required. Patients were excluded if a second relapse occurred. 2) Methadone: Patients on treatment with methadone were included if the treatment was regulated by a maintenance program with stable or decreasing dosification. 3) Benzodiazepines: Therapeutic doses were allowed. Patients with doses over the therapeutic range or a 4-month benzodiazepine use disorder had to be abstinent for 6 months. 4) Cannabis: Patients with daily use and/or high consumption which interfered with the medical follow-up and inability to achieve sustained abstinence. Occasional use was not an exclusion criterion. 5) Tobacco: Abstinence recommended but not required.
- Absence of diagnosis of decompensated schizophrenia or bipolar disorder and chronic mental-organic disorder (dementia, intellectual disability, severe cognitive impairment without social-family support).
- In terms of psychopathological disorders: personality disorders (mainly antisocial disorder), severe depression, eating disorders, and chronic mental-organic disorders (dementia, intellectual disability, severe cognitive impairment with social-family support) were considered as relative contraindications.

The information was gathered according to the DSM-IV definitions and subsequently we adopted the DSM-5 definition of Alcohol Use Disorder which integrates the DSM-IV definitions of alcohol abuse and alcohol dependence into a single disorder.

- American Psychiatric Association (1994). Diagnostic and Statistical Manual of Mental Disorders : DSM-IV. American Psychiatric Publishing, Washington.
- American Psychiatric Association (2013) Diagnostic and Statistical Manual of Mental Disorders. 5th ed. American Psychiatric Publishing, Arlington, VA.

The information regarding the post-LT interventions performed by the local addiction therapists was not available for this study.

Follow-up survey: Survey Information (during visit or by telephonic interview). Telephonic interview for patient or relatives

1) Brief introduction "Hello. We are calling from the Liver and Addictions Unit of Hospital Clínic of Barcelona. We would like to speak with (patient name)".
Alive patients: a) "As you know, Hospital Clínic is a university hospital with a large tradition in research. As a part of our follow-up protocol, we would like to ask you a few questions about your current health condition". b) Brief summary of psychiatric history: we briefly re-assess the psychiatric comorbidity of the patient. i) "Have you had psychiatric treatment for a problem such as depression, anxiety or other before or after LT?" If yes: (1) "What happened? What was the problem? Were you given a diagnosis?" (2) "Were you given any medication? Which one and for how long?" (3) "How are you now with regards to this problem?"
Summary of alcohol, tobacco and substance use after transplantation: We assessed alcohol abstinence time since the transplantation, taking into account quantities, type of beverages and abstinence periods. i) "We understand you know that alcohol consumption was an issue regarding your liver disease and/or the liver transplantation. We would like to know how this subject has evolved since (year of the alcoholic hepatitis episode). Have you drunk any alcohol since the transplantation?" (1) If no: Abstinent (we insist with other questions such as "nothing at all?"). (2) If yes: "Would you mind some questions with regards to this subject; we know it is difficult but the information is important for follow-up": (a) "Since your discharge from the hospital, what has happened with regards to this subject?" (b) "Did you abandon drinking completely or did you resume drinking after transplantation at any time?" If patient says yes: (c) "How long did it take to resume drinking after transplantation?" (d) "What type of alcohol have you been drinking?" (e)"How many drinks per week or maximum alcohol drinks per day? (f) Do you drink now?". i) "Have you smoked any cigarette since the transplantation?" (1) If no: Abstinent (we insist with other questions such as "nothing at all?"), (2) If yes:" (a) Do you currently smoke?". i) "Have you used any substance since the transplantation?", (1) If no: Abstinent (we insist with other questions such as "nothing at all?"), (2) If yes, (a)"what type of substance?", (b)"how often have you been using it?", (c) "are you using it now?".

2) If the patient is deceased: a) We ask to speak with the nearest relative or the person responsible for patient care during the time after discharge from the index admission of the transplantation; we remind the relative the date of the admission. b) "As you know, Hospital Clinic is a university hospital with a large tradition in research. As a part of our follow-up protocol, we would like to ask you a few questions regarding your relative (patient name)": i) "When did he/she die?" ii) "What was the cause of death or final diagnosis?" iii) "Did your relative die in a health care center?" iv) "Do you know if your relative drank any alcohol after being discharged from Hospital Clínic after the liver transplantation?" (Here we explain that any amount of consumption counts). If yes: (1) "Do you remember approximately the amount (e.g. how much) and type of beverages he/she usually drank?" (2) "Do you remember if he/she stopped drinking at any moment after resuming drinking?" If yes: (a) "For how much time did this period last?" (b) "Do you remember how much he/she was consuming after resuming drinking?" (c)"How many drinks per week or maximum alcohol drinks per day?". (v) "Do you know if your relative smoked after being discharged from Hospital Clínic after the liver transplantation?" (vi) "Do you know if your relative used any substance after the liver transplantation?". If yes: (1) "which type of substance?".

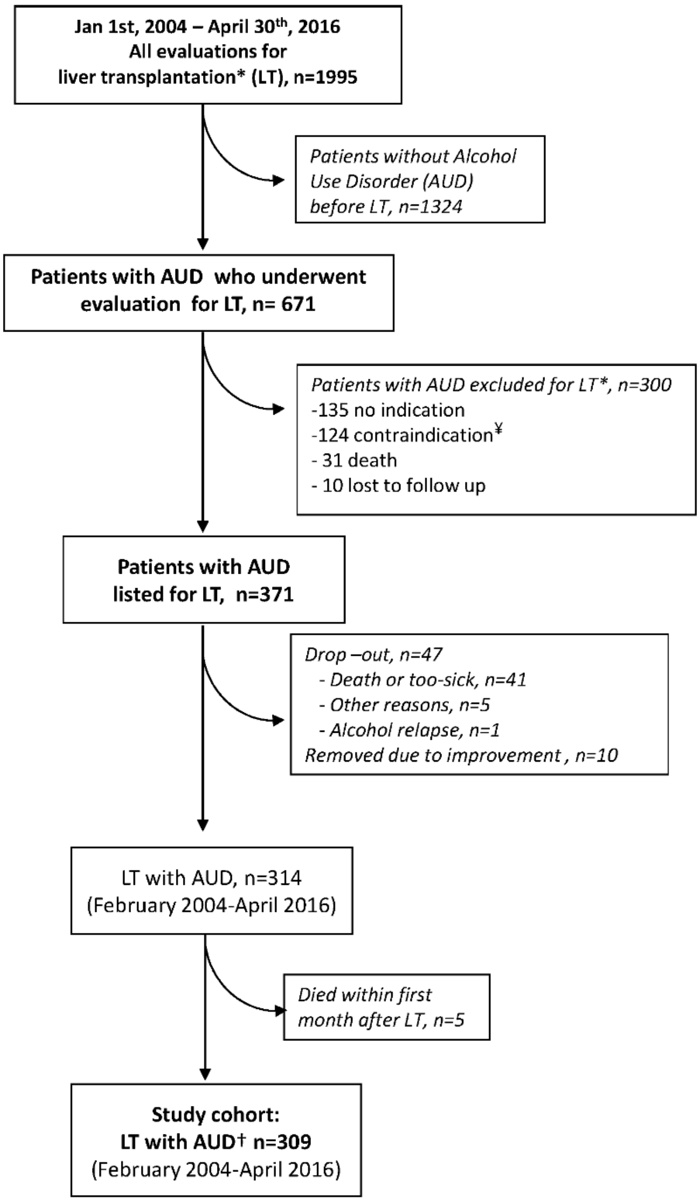

•Data before 2009 includes only patients included after listing.
†138 with concomitant liver disease (136 viral, one autoinmune hepatitis, 1 Corino-Andrade disease)
¥ 12 due to alcohol behaviour; 112 due to medical issues.

Figure S1: recruitment flow-chart.
